# Rainfall Observation Leveraging Raindrop Sounds Acquired Using Waterproof Enclosure: Exploring Optimal Length of Sounds for Frequency Analysis

**DOI:** 10.3390/s24134281

**Published:** 2024-07-01

**Authors:** Seunghyun Hwang, Changhyun Jun, Carlo De Michele, Hyeon-Joon Kim, Jinwook Lee

**Affiliations:** 1 Department of Civil and Environmental Engineering, Chung-Ang University, Seoul 06974, Republic of Korea; hwanghnj@cau.ac.kr (S.H.); cjun@cau.ac.kr (C.J.); hjkim22@cau.ac.kr (H.-J.K.); 2 Department of Civil and Environmental Engineering, Politecnico di Milano, 20133 Milano, Italy; carlo.demichele@polimi.it; 3 Department of Civil and Environmental Engineering, University of Hawaii at Manoa, Honolulu, HI 96822, USA

**Keywords:** rainfall estimation, raindrop sound, frequency analysis, IoT sensor, XGBoost

## Abstract

This paper proposes a novel method to estimate rainfall intensity by analyzing the sound of raindrops. An innovative device for collecting acoustic data was designed, capable of blocking ambient noise in rainy environments. The device was deployed in real rainfall conditions during both the monsoon season and non-monsoon season to record raindrop sounds. The collected raindrop sounds were divided into 1 s, 10 s, and 1 min intervals, and the performance of rainfall intensity estimation for each segment length was compared. First, the rainfall occurrence was determined based on four extracted frequency domain features (average of dB, frequency-weighted average of dB, standard deviation of dB, and highest frequency), followed by a quantitative estimation of the rainfall intensity for the periods in which rainfall occurred. The results indicated that the best estimation performance was achieved when using 10 s segments, corresponding to the following metrics: accuracy: 0.909, false alarm ratio: 0.099, critical success index: 0.753, precision: 0.901, recall: 0.821, and F1 score: 0.859 for rainfall occurrence classification; and root mean square error: 1.675 mm/h, $R^{2}$: 0.798, and mean absolute error: 0.493 mm/h for quantitative rainfall intensity estimation. The proposed small and lightweight device is convenient to install and manage and is remarkably cost-effective compared with traditional rainfall observation equipment. Additionally, this compact rainfall acoustic collection device can facilitate the collection of detailed rainfall information over vast areas.

## 1. Introduction

Acoustic observations based on microphones have been consistently applied across various fields for numerous purposes. For instance, monitoring systems have been developed to detect equipment operation status and damage by capturing the sounds of operating machinery in automated manufacturing processes [1]. Moreover, acoustic observations based on vocalizations, such as those of clusters of tropical animals, have been used to observe the distribution characteristics of insect species within the order Orthoptera [2]. Notably, the use of cost-effective recording equipment and small data sizes for acoustic observations has proven highly efficient in observing vast regions with limited resources, thereby contributing to the advancement of research fields such as soundscape and bioacoustics. Examples include the analysis of acoustic patterns produced during the mating period of humpback whales to analyze population size and behavioral patterns [3] and the real-time collection of natural acoustics to analyze species distribution in target areas [4].

Recent advancements in artificial intelligence (AI) have led to a growing trend of integrating acoustic observations with AI techniques. Loey and Mirjalili [5] constructed a model for identifying COVID-19 patients based on cough sounds. The authors explored the applicability of six deep learning neural networks (GoogleNet, ResNet18, ResNet50, ResNet101, MobileNetv2, NasNetmobile) and compared the performance of these models to identify the optimal model. Domingos et al. [6] aimed to detect illegal fishing vessels in coastal areas by developing a model to determine the presence and type of ships based on underwater sounds. They classified the input data using the constant Q transform spectrogram, Gammatone spectrogram, Mel spectrogram, and their combinations and compared the performance of VGGNet and ResNet18 in ship classification. Ekpezu et al. [7] developed a model for classifying four types of natural disasters: earthquakes, tornadoes, tsunamis, and volcanic eruptions, using acoustic data, comparing the classification performance of a convolutional neural network (CNN) and long short-term memory.

In the domain of rainfall observation, recent studies have used a range of novel sensors to estimate the rainfall intensity and drop size distribution and predict precipitation. Notable examples include rain intensity observations based on a piezoelectric sensor equipped with a Unimorph piezo disc [8], rainfall intensity estimation using LiDAR point cloud features derived from LiDAR sensors integrated into autonomous vehicles [9], precipitation estimation through cloud image capture and analysis [10], and determination of rainfall intensity and drop size distribution using infrared footage from surveillance cameras [11].

As discussed, acoustic observations have shown remarkable efficiency in observing vast areas with limited resources, rendering them highly applicable to rainfall observation. Several researchers have used acoustic data to observe rainfall. Dunkerley [12] recalculated rainfall duration based on raindrop sounds to mitigate errors associated with tipping-bucket rain gauges with different time resolutions. Experiments were conducted with tipping-bucket rain gauges having time resolutions of 5 min, 15 min, and 1 h, and rainfall intensity differences of up to 300% or more were observed with the corrected rainfall duration. Research has been conducted to identify differences in rainfall acoustics based on rainfall intensity through spectral analysis, with the goal of estimating the rainfall intensity accordingly. Avanzato and Beritelli [13] investigated the differences in rainfall acoustics according to rainfall intensity by simultaneously using a tipping bucket rain gauge and a recording device to collect both acoustic and intensity data. Using PRAAT software, the authors analyzed changes in acoustic features such as spectrogram, formants, pitch, and intensity in relation to rainfall intensity, aiming to estimate rainfall intensity based on these acoustic characteristics. Nakazato et al. [14] aimed to develop a technology for generating computer graphic (CG) animations of rainfall scenes that naturally align with acoustic data. The authors analyzed two characteristic values—maximum frequency and cumulative relative frequency—from the Fourier transform results of rainfall acoustics in relation to changes in rainfall intensity. Based on these characteristic values, they proposed a relational equation for estimating rainfall intensity from acoustic data and developed a technique for generating CG animations of rainfall scenes.

Furthermore, numerous previous studies have been conducted on observing rainfall based on acoustic signals. Nystuen [15] and Nystuen and Amitai [16] estimated the drop size distribution based on raindrop sounds collected beneath the water surface. The authors analyzed the frequency domain characteristics of the shock waves generated when the raindrops reached the water surface, considering different rainfall types and drop sizes. The results were compared with the drop size distribution observed using a disdrometer, and eventually, the rainfall intensity and drop size distribution were estimated. Furthermore, underwater raindrop sounds and machine learning have been combined to estimate rainfall intensity. Trucco [17] compared the performances of the linear discriminant analysis, logistic regression, support vector machine, and random forest methods in estimating the rainfall intensity based on underwater raindrop sounds. Bedoya et al. [18] estimated the rainfall intensity based on raindrop sounds collected in tropical rainforests. The authors extracted specific frequency domain ranges primarily containing acoustic signals of rainfall occurring in vegetated regions and aimed to determine the occurrence of rainfall, while classifying and estimating the rainfall intensity based on the average power spectral density. Guo et al. [19] classified the rainfall intensity into three levels by collecting acoustic data of raindrops falling on umbrellas using smartphones. The authors first differentiated between the presence and absence of rainfall based on the collected acoustic data and then identified whether the sound originated from raindrops colliding on umbrellas. Finally, they classified the rainfall intensity into three levels based on the sounds of the raindrops. The authors considered several frequency characteristics for rainfall detection, including the minimum, median, and average amplitude, root mean squared error-low, spectral similarity, spectral decrease, amplitude of middle frequency, and cut-off frequency. For the recognition of raindrops hitting an umbrella, they analyzed the average absolute amplitude, average zero-crossing rate, Mel-frequency cepstral coefficient, and spectral centroid. To estimate the rainfall intensity, they evaluated the maximum amplitude, energy-low, spectral roll-off, and spectral slope. Wang et al. [20] classified the rainfall intensity into five levels using acoustic data collected from surveillance cameras. The authors extracted 16 input features from five spectrograms (chroma, tonal centroid features, spectral constant features, Mel-frequency cepstral coefficients, and log-Mel spectrograms) and their combinations. Additionally, they applied three different CNN architectures with varying layer structures and compared their performances in rainfall intensity classification.

However, previous studies using acoustic data for rainfall observation have encountered their own limitations:

First, in the case of rainfall acoustics recorded underwater [15,16,17], the surfaces on which raindrops collide are limited to the water surface, resulting in a lower variability compared to rainfall acoustics collected in terrestrial environments with relatively diverse surface conditions. Comparing the characteristics of the collision sounds generated by water droplets falling on different surface materials [21], it is evident that the acoustic properties vary significantly depending on the material and environment of the surface on which the water droplets fall. Consequently, methods for estimating the rainfall intensity based on underwater acoustics may face challenges when applied to terrestrial rainfall acoustics, which involve relatively diverse surface conditions. In this study, rainfall acoustics collected at Chung-Ang University in Seoul, South Korea, were analyzed. By attempting to develop techniques for rainfall intensity estimation capable of accommodating high variability in surrounding environments and diverse forms of noise (e.g., human speech, wind noise, etc.), this study aimed to enable rainfall intensity estimation even in environments prone to various types of noise.

Second, in the collection of acoustic data, previous studies utilizing devices such as open-type microphones [18] or smartphones [19] to gather and utilize external environmental sounds have encountered issues due to the sensitivity of acoustic data to changes in the environment. For example, the case analyzed by Bedoya et al. [18], which examined rainfall acoustics in tropical rainforest regions, may face difficulties when applied to urban areas. In the study by Guo et al. [19], the researchers attempted to address this issue by using only rainfall acoustics collected under an umbrella. However, this approach also exhibited high variability in the rainfall acoustics depending on the material and dimensions of the umbrella. In this study, to mitigate the high variability in the acoustic data caused by changes in the external environment, an external housing controlled with specific materials and dimensions was considered. The microphone was fixed inside the waterproof enclosure, and the setup was designed to dominantly collect the acoustics generated by raindrops colliding with the surface of the enclosure, ensuring consistent acoustic collection despite changes in the external environment. We assessed the effectiveness of our approach to mitigate variability caused by changes in the surrounding environment and evaluated the generalizability of the algorithm proposed in this study. This was achieved by testing the algorithm with acoustic signals collected in entirely different environments from those used to develop the rainfall detection and intensity estimation algorithm.

Third, using rainfall intensity data based on the subjective judgment of the individuals collecting the data, rather than precise rainfall observation instruments, can lead to discrepancies in the perceived rainfall intensity for the same event, thereby reducing the accuracy of the developed model. In Guo et al. [19], the rainfall intensity of the collected rainfall acoustics was classified into three levels based on the subjective judgment of the observer rather than precise instruments. This approach is likely to produce less objective results compared to using the rainfall intensity measured by precise observation instruments. In this study, rainfall intensity data observed by an optical disdrometer, PARSIVEL, installed adjacent to the acoustic data collection device were used for validation, ensuring objective and reliable results. The PARSIVEL, used in this study to generate reference values for the rainfall intensity, has already been proven to be highly accurate in previous studies [22].

Lastly, most previous studies have attempted to classify rainfall intensity into a few discrete categories, with limited emphasis on a quantitative estimation of the rainfall intensity. Studies attempting to quantitatively estimate the rainfall intensity are relatively scarce. This study aims to derive quantitative measures of the rainfall intensity based on rainfall acoustics, with the goal of obtaining more precise rainfall information.

Before estimating the rainfall intensity based on rainfall acoustics, it is essential to comprehend the characteristics of the acoustics produced by rainfall particles. Based on prior research that analyzes the behavior of individual rainfall particles and the acoustic phenomena generated by the collision of single water droplets, we aimed to design a research methodology capable of sensitively capturing changes in rainfall acoustics corresponding to variations in rainfall intensity.

As the rainfall intensity increases, larger rain particles are generated [23]. Due to their larger diameter, water droplets of significant size fall more rapidly [24]. Consequently, larger rain drops in heavy rainfall events exhibit higher terminal velocities [25,26]. Prokhorov and Chashechkin [27] examined the characteristics of sound generated under varying speeds of water droplets falling onto the water surface. In terms of the initial sound produced upon the collision of a water droplet with the water surface, it has been established that a higher frequency band sound is produced when the water droplet falls at a higher speed.

Beacham et al. [21] analyzed the acoustic signal behavior induced when water droplets fall onto surfaces of four different shapes: deep water surface, thin film of water on a smooth aluminum surface, dry aluminum surface, and masonry brick surface, based on indoor experiments. Among these, the dry aluminum surface and thin film of water on smooth aluminum surfaces exhibit high similarity to the characteristics of rainfall acoustics captured by rainfall acoustic collection devices deployed in actual rainfall environments. The waterproof enclosure of the plastic material utilized in the rainfall acoustic collection device features a smooth surface and flat upper structure. Consequently, as rainfall persists, a thin water film is created by the water droplets adhering to the upper surface. According to the experimental results of Beacham et al. [21], when dry aluminum is impacted by drops, the liquid is dispersed into a thin disc. In this case, the resulting acoustic signals are uniformly distributed within the range of 0 to 25 kHz. However, notably weaker acoustic signals occur compared with those in the other three cases. In contrast, when water droplets fall onto the thin film of water on a smooth aluminum surface, sound is generated, which can be distinguished by two distinct acoustic characteristics: sound produced by the collision of water droplets with the surface and sound generated during the bursting of air bubbles formed beneath the surface during collision. The second sound exhibits a lower frequency range compared with that of the first sound. Such characteristics are remarkably similar, even in deep water surfaces.

Based on the notable resemblance in the collision acoustics of single water droplets falling onto deep water surfaces and thin films of water layers, as evidenced by the findings of Beacham et al. [21], it is inferred that the characteristics of rainfall acoustics on water surfaces, where a higher rainfall intensity progressively generates higher-frequency acoustics, may similarly apply to rainfall acoustics produced by thin films of water layers, as observed in this study. Given the anticipated increase in high-frequency rainfall acoustics with increasing rainfall intensity, this study aimed to ensure heightened sensitivity to changes in the rainfall intensity by considering feature weights that emphasize the high-frequency domain.

In this study, we compared the collected raindrop sounds with rainfall intensity data acquired using PARSIVEL to establish a rainfall intensity estimation model based on acoustic data. The raindrop sound data were subjected to short-time Fourier transform (STFT) to extract four frequency domain characteristics. These four characteristics served as input data for a preliminary binary classification process based on extreme gradient boosting (XGBoost) to determine the presence or absence of rainfall. Subsequently, we quantitatively estimated the rainfall intensity for the period during which the rainfall was judged to have occurred. During the rainfall intensity estimation process, we divided the collected raindrop sounds into segments of 1 s, 10 s, and 1 min and compared the estimation performance for each of these segments. This research provides a non-discrete and quantitative approach to estimate the rainfall intensity using terrestrial raindrop sounds, addressing several limitations observed in previous studies and contributing to the advancement of acoustic-based rainfall observation techniques.

## 2. Materials and Methods

**Figure 1:**
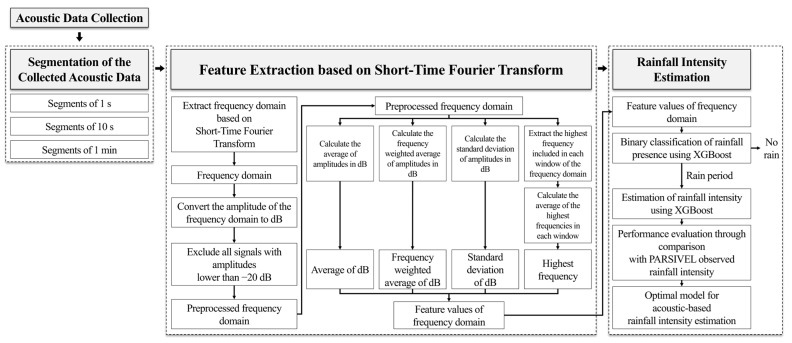
Process flow of this study.

The research flow is illustrated in Figure 1. Firstly, we designed an acoustic data collection device using a condenser microphone and Raspberry Pi system and collected raindrop sounds. Only data collection was conducted with the Raspberry Pi as an acoustic data collection device, with all subsequent processes carried out on a separate workstation. The collected raindrop sounds were segmented into three intervals with lengths of 1 min, 10 s, and 1 s. Subsequently, the STFT was applied to each segment to extract the spectrogram in the frequency domain. The magnitude of the spectrogram was converted to decibel (dB) units. To enhance the distinction between the acoustic signals of high and low rainfall intensities, all frequency regions not exceeding −20 dB were adjusted to −30 dB. From the final spectrogram, four frequency domain feature values were extracted: average of dB, frequency-weighted average of dB, standard deviation of dB, and highest frequency. These values were subsequently used to develop a machine learning model for rainfall detection and intensity estimation. The dataset of the extracted four feature values was labeled with the rainfall intensity observed by PARSIVEL at the same time as the segments. Using the entire dataset, a binary classification model for rainfall detection was trained and validated. The dataset for the rainfall intensity estimation model was composed of the periods identified as when rainfall occurred according to the validation results of the binary classification model. The accuracy of the final estimated rainfall intensity was evaluated by comparing it with the rainfall intensity observed using PARSIVEL. Detailed explanations for each stage are presented in Section 2.1, Section 2.2, Section 2.3 and Section 2.4 and the Python (version 3.9.19) code utilized in data collection, frequency analysis, and construction of machine learning models for this study is provided alongside it.

### 2.1. Acoustic Data Collection Device Based on Internet of Things (IoT) Sensor

**Figure 2:**
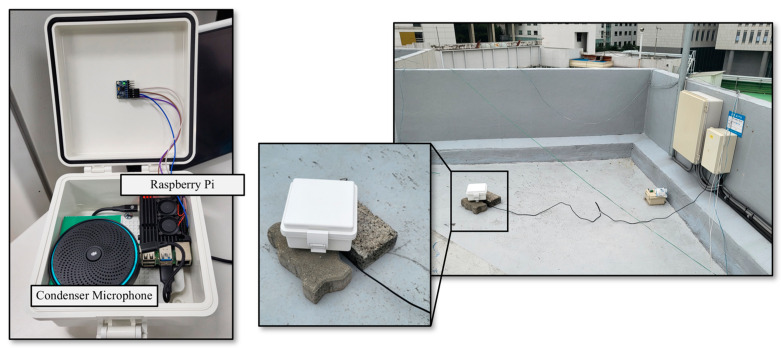
Acoustic data collection device.

To collect raindrop sounds generated during rainfall, we constructed an acoustic data collection device by installing a condenser microphone and Raspberry Pi inside plastic waterproof housing. Raspberry Pi is an ultra-small onboard computer with powerful performance, which can be integrated with a microphone to function as an efficient recording device. In this study, a Raspberry Pi 4 Model B with 8 GB of RAM was utilized, which ran the Raspberry Pi OS. Figure 2 shows the appearance and installation configuration of the constructed device.

The waterproof plastic enclosure effectively prevents rainwater from infiltrating the condenser microphone and Raspberry Pi, thereby enabling the deployment of the acoustic data collection device under rainy conditions. Furthermore, it ensures the collection of consistent rainfall acoustics, regardless of the surrounding environment. In a rainfall environment, acoustic signals are generated when raindrops collide with the surrounding medium. The type of medium can affect the acoustic data produced. In other words, depending on the environmental conditions around the device installation location, the collected acoustic data can vary, even in the same rainfall environment. This variability can act as significant interference when using acoustic data for rainfall intensity estimation. In this study, we mitigated the influence of background noise originating from the ambient environment by embedding a condenser microphone and Raspberry Pi within waterproof plastic housing. This setup effectively blocked background noise, predominantly capturing acoustic data generated when raindrops impacted the plastic housing, ensuring consistent acoustic data collection regardless of the installation environment. The Raspberry Pi, connected to the condenser microphone, formed an embedded system for acoustic data collection, storing 60 s segments of acoustic signals at a sampling rate of 44.1 kHz.

**Figure 3:**
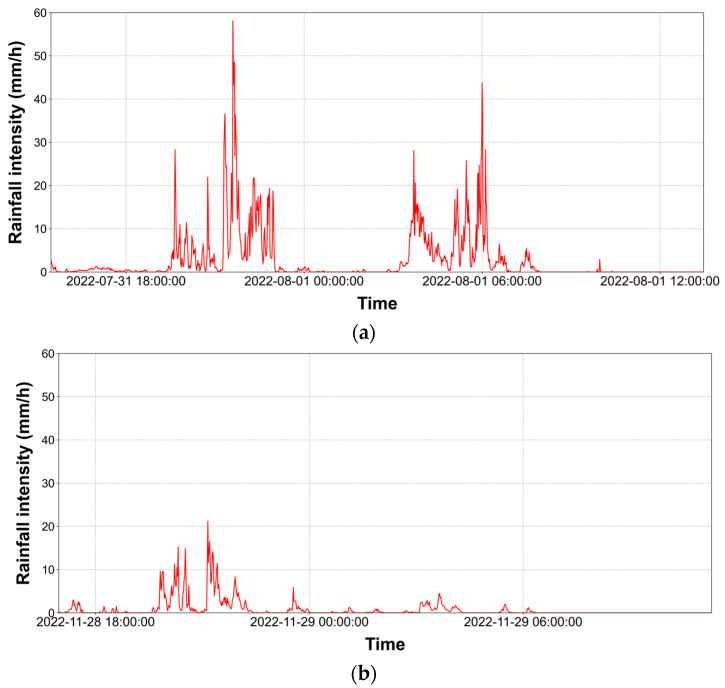
Rainfall events observed during the research period: (**a**) monsoon season (31 July 2022, to 1 August 2022); (**b**) non-monsoon season (28 November 2022, to 29 November 2022).

The acoustic data collection device was installed on the rooftop of the Bobst Hall at Chung-Ang University, located in Seoul, South Korea (37°20′13″ N, 126°57′27″ E), to collect acoustic data in rainfall conditions. The Korean Peninsula has a monsoon climate with rainfall concentrated in June, July, and August. In this study, we focused on two periods: the monsoon season (31 July 2022, to 1 August 2022) and non-monsoon season (28 November 2022, to 29 November 2022). Table 1 presents the data used in this study, while Figure 3 illustrates the rainfall intensity in minutes observed by the PARSIVEL device installed at the same location as the acoustic data collection device during the study period.

The maximum rainfall intensity and cumulative precipitation in the monsoon season are 58.1 mm/h and 58.9 mm, respectively, with the corresponding values in the non-monsoon season being 21.3 mm/h and 14.4 mm.

**Figure 4:**
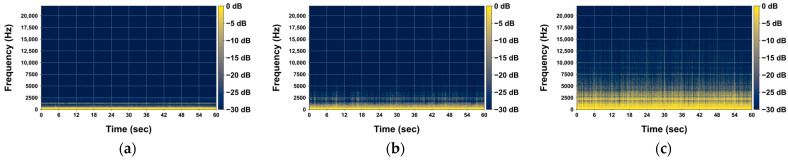
Example of 1 min length spectrograms of collected acoustic data: (**a**) 29 November 2022 10:10 a.m. (No rain); (**b**) 31 July 2022 08:17 p.m. (5 mm/h); (**c**) 31 July 2022 09:35 p.m. (58 mm/h).

Figure 4 illustrates examples of the rainfall acoustics collected using the developed acoustic data collection device. Figure 4a presents the acoustic data collected during a no-rain period, while Figure 4b shows the acoustic data for a period with a recorded rainfall intensity of 5 mm/h. Figure 4c depicts the acoustic data corresponding to the period with the highest recorded rainfall intensity during the study period, which was 58 mm/h. As inferred from previous studies, it has been confirmed that an increase in rainfall intensity leads to the emergence of high-frequency acoustic signals. The frequency analysis results for the non-rainfall period, as shown in Figure 4a, indicate that acoustic signals are present only within the frequency range below 2500 Hz. However, in the acoustic signals of rainfall with an intensity of 5 mm/h (Figure 4b), acoustic signals begin to appear within the frequency range of 2500 Hz to 5000 Hz. Furthermore, during the period with the highest rainfall intensity within the study period (Figure 4c), numerous acoustic signals with frequencies above 5000 Hz were also detected.

**Figure 5:**
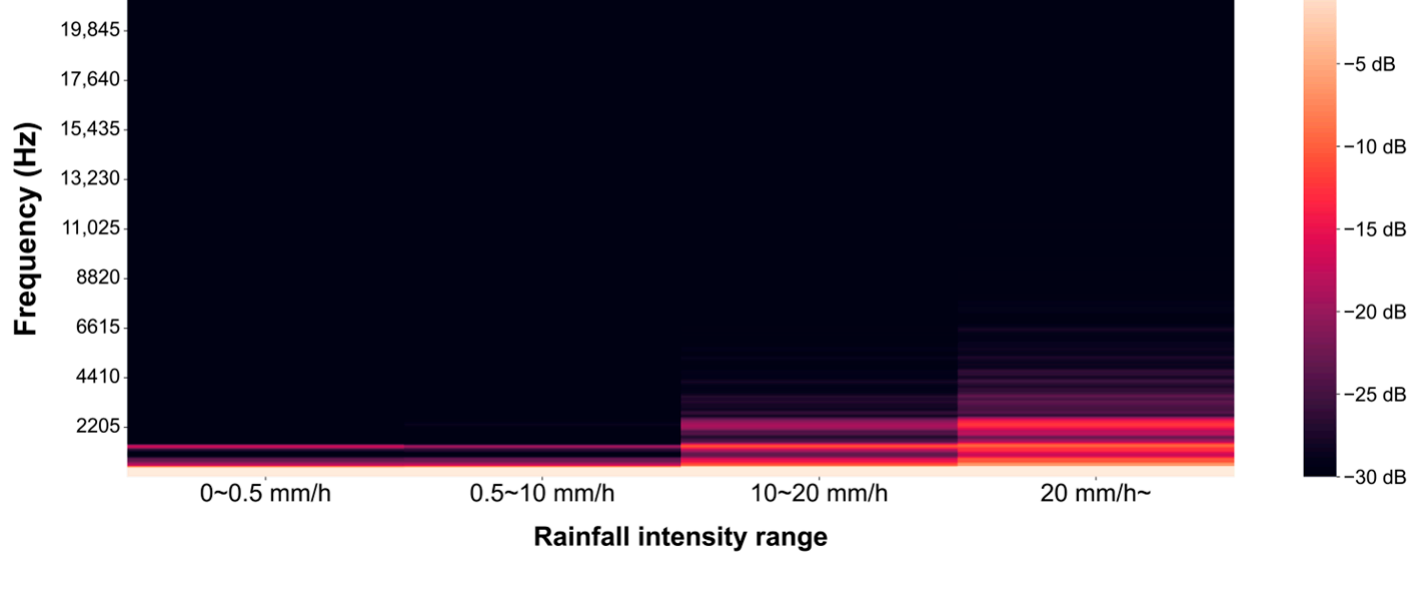
Average of dB values by frequency according to rainfall intensity.

These characteristics were observed throughout the collected rainfall acoustic data. Figure 5 illustrates the average of dB values across frequency domains for rainfall acoustics collected over the entire study period, segmented by rainfall intensity ranges. Compared to the periods of no rain with recorded rainfall intensities ranging from 0 to 0.5 mm/h, it was observed that additional high-frequency acoustics occurred during the remaining periods of rainfall occurrence. Furthermore, as the rainfall intensity increased, acoustic signals with progressively higher frequencies were included.

### 2.2. Observation Data from Disdrometer

**Figure 6:**
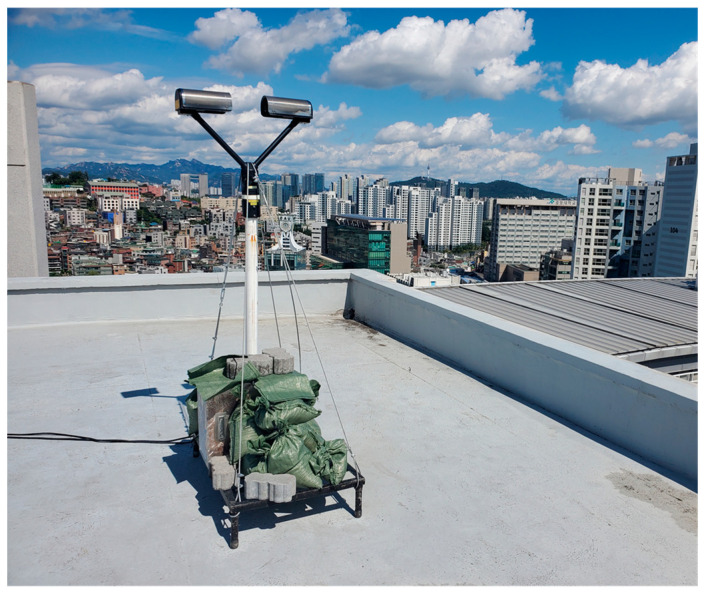
A depiction of the PARSIVEL installation implemented in this study.

In this study, rainfall intensity data obtained from a PARSIVEL, positioned near the acoustic data collection device, were utilized for validation purposes (Figure 6). PARSIVEL is a ground-based meteorological instrument that measures the diameter and fall velocity of precipitation particles (raindrops, snowflakes, hailstones). This information, including raindrop size, is used to estimate quantitative precipitation and elucidate the microphysical properties and development mechanisms of precipitation systems [28].

**Figure 7:**
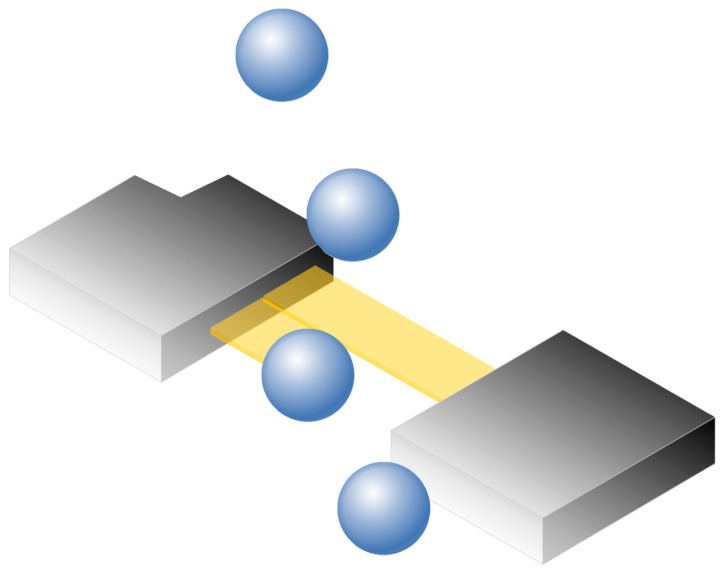
Functional principle of the PARSIVEL disdrometer.

**Figure 8:**
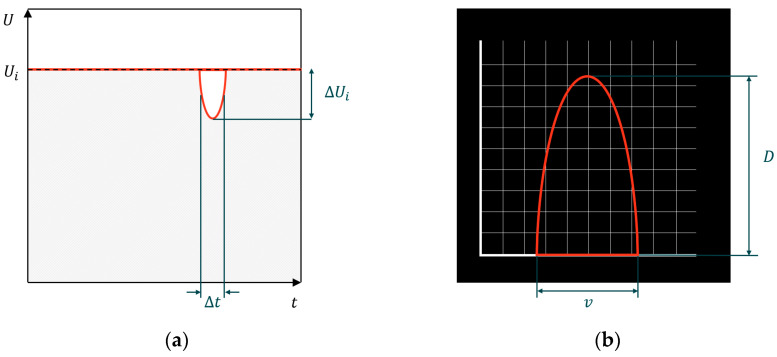
(**a**) When a particle passes through the beam within the measurement area, it causes a change in the signal. (**b**) The particle’s size is indicated by the extent of signal dimming, and the fall velocity is determined from the duration of the signal.

PARSIVEL utilizes a laser-based optical sensor to measure the diameter and fall velocity of precipitation particles. A laser beam emitted from the transmitter is continuously received by the receiver. When precipitation particles pass through the laser beam, the interruption of the laser signal is observed to determine the size and fall velocity of the particles (Figure 7) [29]. The diameter and velocity of the particles are calculated based on the duration it takes for the particles to traverse the laser beam and the attenuation of the laser intensity during this passage (Figure 8).

The PARSIVEL employed in this study is the second version produced by the German manufacturer OTT, which demonstrates enhanced accuracy in detecting smaller particles compared to its predecessor. Detailed specifications of the PARSIVEL used in this study are presented in Table 2.

### 2.3. STFT-Based Feature Extraction

Various acoustic signals in our surroundings, such as speech, music, and noise, are composed of complex waveforms resulting from the superposition of sinusoidal waves, each with their own amplitude, frequency, and phase displacement [30]. The Fourier transform can be used to analyze the frequencies of individual sinusoidal components constituting the complex waveform of an acoustic signal by transforming it from the time domain to the frequency domain. Equation (1) represents the fundamental equation for the Fourier transform, f(α), of a function f(x), where x and α are parameters related to time and frequency, respectively [31]. When the frequency parameter α aligns with the periodicity of the function f(x), the value of f(α) increases sharply. The magnitude of f(α) can be used to discern the frequency characteristics of the periodic components of f(x).

(1) $$f\left(\alpha \right)=\int_{-\infty }^{\infty }f\left(x\right)e^{i\alpha x}dx$$

The Fourier transform is traditionally applied to continuous functions over an infinite time range. However, methods exist to implement it for discrete digital signals. These methods, referred to as the discrete Fourier transform (DFT), allow for the representation of discrete frequency characteristics within sampled time domain signals, such as acoustic signals. The DFT is fundamentally computed over a windowed, finite time range. It is calculated for the *n*th time sample $x_{n}$ and the *k*th frequency sample $\alpha_{k}$ within specified time intervals and frequency ranges, as described by the following equation [32].

(2) $$f\left(\alpha_{k}\right)=\sum_{n=0}^{N-1}f\left(x_{n}\right)e^{i\alpha_{k}x_{n}},\;\;k=0,\;1,\;2,\;\ldots ,\;N-1$$

Despite the effectiveness of the Fourier transform in examining the frequencies of the individual sinusoidal components constituting complex acoustic signals, it sacrifices the time information during the transformation of an acoustic signal from the time domain to the frequency domain. Consequently, Fourier transform may be inadequate when analyzing signals with temporal variations. The concept of STFT has been introduced to overcome this problem. The STFT can be derived by iteratively extracting short-duration windows from the time domain signal and applying the DFT. Signals confined within finite window intervals undergo discretization into the frequency domain via the application of DFT, facilitating the extraction of a continuous frequency domain representation concerning the time domain by shifting these windows at predetermined intervals. Ultimately, by concatenating the sequentially extracted frequency domains along the time axis, STFT computation is feasible. This approach can effectively enable the observation of temporal variations in frequency domain characteristics. STFT preserves both time and frequency information, facilitating the analysis of time-varying signals [33]. This can be expressed mathematically as follows [34]:

(3) $$f_{n}\left(e^{i\alpha_{k}}\right)=\sum_{x=-\infty }^{\infty }w\left(n-x\right)f\left(x\right)e^{i\alpha_{k}x}$$

where $f_{n}\left(e^{ia_{k}}\right)$ is the STFT of *n*th window and $w\left(n-x\right)$ is the window function.

In this study, we used the STFT to analyze the frequency characteristics of the collected acoustic data to estimate the rainfall intensity. For the computation of the STFT on an audio signal sampled at 44,100 Hz, the window size was set to 441, equivalent to a duration of 0.01 s. The spacing between adjacent windows was set to 512. In general, raindrops exhibit high variability as they fall, resulting in diverse raindrop sound patterns. To ensure accurate rainfall intensity estimation, this variability must be mitigated by accumulating long-duration acoustic data. However, using excessively long acoustic data may limit the available data for the entire research period, potentially affecting the training performance of machine learning models. Additionally, the use of shorter acoustic data segments can lead to a higher temporal resolution in the final estimated rainfall intensity. Therefore, the length of the acoustic data used for rainfall intensity estimation should be as short as possible while ensuring sufficient accuracy, necessitating the identification of the optimal length of acoustic data for rainfall intensity estimation. We divided the acoustic data into 1 s, 10 s, and 1 min intervals and compared the corresponding performances to identify the segment that could ensure the best balance between the accuracy and temporal resolution for rainfall intensity estimation.

The acoustic data with different segment lengths were subjected to STFT to extract the frequency domain characteristics. Subsequently, the magnitudes of the spectrogram-extracted frequency domains were converted into dB units. To convert magnitudes into dB units, the Python library Librosa was used. In general, dB units are logarithmic values for amplitude, set within a range based on the maximum amplitude value present in the spectrogram. When the maximum amplitude value in a specific spectrogram is *K*, the amplitude is converted to dB units using the following formula [35]:

(4) $$dB\;units=Max\;(20\;*\;{log}_{10}(amplitude),\;20\;*\;{log}_{10}(K)-80)$$

In analyzing the spectrogram, we aimed to exclude components with weak periodicity by replacing dB with a consistent value for frequency ranges that had dB values below a specific criterion. After several trials, we determined suitable criteria and replacement values. When the magnitude did not exceed −20 dB, we uniformly replaced all values with −30 dB. These thresholds are employed to delineate between the acoustic characteristics of heavy and light rainfall intensities. They are implemented to effectively discern the robust high-frequency components generated by heavy rainfall from the subtle high-frequency elements typically observed in light rainfall. By utilizing thresholds, the inclusion of subtle high-frequency components is mitigated, thereby enhancing the distinction between the acoustic profiles of heavy rainfall, characterized by pronounced high-frequency features, and those of light rainfall. Ultimately, four feature values (average of dB, frequency-weighted average of dB, standard deviation of dB, and highest frequency) were extracted from the frequency domain for determining rainfall presence or absence and estimating its intensity.

The application of STFT to the acoustic signal resulted in a continuous series of Fourier transform outcomes for each window. In this context, the average of dB values across all frequency ranges for all windows were used to derive the average of dB:

(5) $$Average\;of\;dB=\frac{\sum_{i=1}^{n}\sum_{j=1}^{m}a_{ij}}{nm}$$

where *n* is the number of frequency bins sampled at intervals of 44.1 Hz across the entire frequency range of 0 to 22,050 Hz, while *m* is the count of windows sampled at intervals of approximately 0.011 s over the entire duration of the dataset. $a_{ij}$ signifies the dB value of the frequency domain corresponding to the frequency range of (22,050−i·44.1) Hz sampled within the window at (0.011·j) seconds.

To increase the sensitivity in detecting changes in the characteristics of the high-frequency domain as the rainfall intensity increases, a weighting mechanism was applied to prioritize the high-frequency domain. This involved the assignment of weights from 0 to 499 to the 500 segmented frequency domains, starting from the low-frequency range and extending to the high-frequency range. The frequency range with weak signals involved values of −30 dB or close to −20 dB, and these values gradually increased as the signal strength increased and became positive. However, when the frequency range with large signals approached values closer to 0 dB, the applied weighting was offset. To solve this problem, we added 30 to the dB values of all frequency regions. The following expression was used to calculate the frequency-weighted average of dB values:

(6) $$Frequency-weighted\;average\;of\;dB=\frac{\sum_{i=1}^{n}(500-i)\sum_{j=1}^{m}\left(a_{ij}+30\right)}{nm}$$

where *n*, *m*, and $a_{ij}$ are consistent with Equation (5).

The standard deviation of dB values was defined as the standard deviation of dB values across all frequency ranges for all windows, calculated as follows:

(7) $$Standard\;deviation\;of\;dB=\sqrt{\frac{\sum_{i=1}^{n}\sum_{j=1}^{m}{\left(a_{ij}-\bar{a}\right)}^{2}}{nm-1}}$$

where *n*, *m*, and $a_{ij}$ are consistent with Equation (5), while $\bar{a}$ denotes the average of dB values across all windows and frequency domains extracted from the spectrogram encompassing the entire frequency range.

As mentioned previously, the experimental data demonstrated that as rainfall intensity increases, the frequency distribution of raindrop sounds expands into the high-frequency domain. Therefore, the highest frequency contained in the raindrop sounds was extracted as a feature value. For the 500 frequency-domain ranges, indices ranging from 0 to 499 were assigned, starting from the low-frequency range to the high-frequency range. The index of the highest frequency domain containing acoustic signals was extracted for each window. The average of these maximum frequency values across all windows was used as the highest frequency value, determined as follows:

(8) $$Highest\;frequency=\frac{1}{n}\sum_{j=1}^{m}Max(row\;indices\;of\;frequency\;range>-30\;dB)$$

### 2.4. Rainfall Intensity Estimation Based on XGBoost

XGBoost, an improved version of the gradient boosting machine (GBM) utilizing gradient boosting-based ensemble techniques, was developed to enhance the computational speed of the traditional GBM [36]. In boosting-based ensemble methods, multiple weak learners, which do not perform well individually, are combined to achieve strong predictive performance [37]. Gradient boosting involves sequentially adding weak learners that predict the residuals between predicted and actual values, ultimately improving accuracy by adding the sum of the final predicted residuals to the initial predictions.

**Figure 9:**
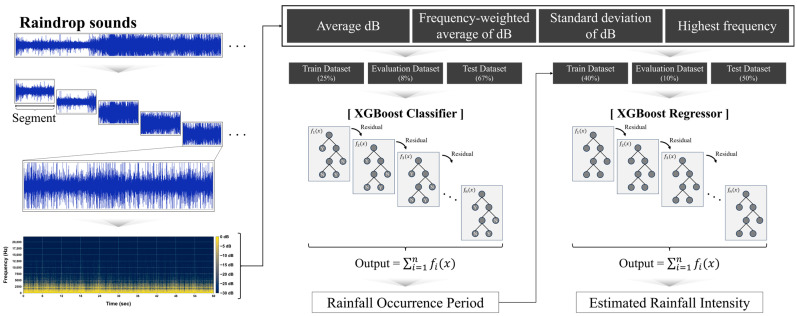
XGBoost-based model for rainfall detection and intensity estimation.

In this study, we applied XGBoost to build a binary classification model to detect the presence or absence of rainfall, along with a rainfall intensity estimation algorithm. Figure 9 presents the XGBoost-based model utilized in this study for binary classification of rainfall presence and intensity estimation. To construct a dataset for training and validating the machine learning model, the presence and intensity of rainfall were labeled based on the rainfall intensity observed by PARSIVEL. Subsequently, a binary classification model for the presence of rainfall was developed using the entire dataset. And a regression model for estimating rainfall intensity was constructed using the dataset corresponding to the periods identified as having rainfall based on the validation results of this binary classification model.

For binary classification, one-third of the entire research period was used as the training dataset, with the remainder serving as the test dataset. Additionally, 25% of the training dataset was allocated for an evaluation dataset to prevent overfitting. The evaluation dataset is not utilized for parameter optimization of the machine learning model. Instead, it is exclusively used to provide continuous performance evaluation results during the training process. Training was halted when no further improvement in performance on the evaluation dataset was observed. This is designed to prevent overfitting, where the model’s parameters are optimized to enhance accuracy in the training dataset but may lead to decreased performance in the evaluation dataset. By ensuring that the test dataset is not used during the training process through the evaluation dataset, the integrity of the validation results for the test dataset is maintained, while simultaneously constructing a generalized model. After the model training was completed, the binary classification model for rainfall presence was validated using the test dataset. Based on the validation results, the periods identified as having rainfall were extracted and used as the dataset for the rainfall intensity estimation model.

For rainfall intensity estimation, half of the dataset, corresponding to periods with rainfall, was used as the training dataset, with the remaining portion serving as the test dataset. In this case, 20% of the training dataset was allocated for the evaluation dataset. During the training process of the rainfall intensity estimation model, the parameter of the model was optimized using the training dataset, and training was halted when performance on the evaluation dataset ceased to improve. After the training was completed, the performance of the rainfall intensity estimation model was validated using the test dataset.

In the process of model construction, because the input data for the regression model for rainfall intensity estimation was derived from the validation results of the classification model for rainfall detection, a higher proportion of the test data in the classification model was allocated to ensure the availability of as much input data as possible for the regression model. The acoustic data utilized in this study corresponded to monsoon and non-monsoon seasons. Concerns arose regarding potential differences in the characteristics of training and test data owing to variations in rainfall amounts between monsoon and non-monsoon seasons when dividing them chronologically. To address this issue, an even sampling of training and test data across the entire period was conducted to mitigate potential discrepancies stemming from differences in rainfall amounts between monsoon and non-monsoon seasons.

Optuna, a Python library, was used to identify the optimal hyperparameters for both the XGBoost classifier and regressor [38]. Among the various sampling methods offered by Optuna for hyperparameter optimization, we used the tree-structured Parzen estimator (TPE) sampler for optimization. The TPE sampler, based on the Gaussian distribution of hyperparameter values and their corresponding loss, determines the next hyperparameter value for the following iteration by leveraging the results of previous iterations. This method is faster than the grid search or random search methods [39]. The optimal hyperparameters, determined through 1000 search iterations, were used in the training process.

### 2.5. Assessment Method

When rainfall intensity is estimated using segments of 1 s, 10 s, and 1 min in length, the final estimated rainfall intensity also possesses a temporal resolution of 1 s, 10 s, and 1 min, respectively. However, the observational data from PARSIVEL, used for validation in this study, have a temporal resolution of either 1 min or 10 s. To evaluate the results of the binary classification for rainfall detection and the final estimated rainfall intensity, we converted the PARSIVEL data produced at 10 s and 1 min intervals into datasets with temporal resolutions of 1 s, 10 s, and 1 min. For the data collected at 10 s intervals, we assumed a constant rainfall intensity throughout each 10 s period for each observation, converting it into 1 s interval data. Additionally, we averaged six 10 s interval rainfall intensities collected over a 1 min period to convert it into 1 min interval data. Similarly, for the data collected at 1 min intervals, we assumed a constant rainfall intensity throughout the 1 min period for each observation, converting it into datasets with 1 s and 10 s intervals.

The classification performance was evaluated using several metrics, i.e., the accuracy, false alarm ratio (FAR), critical success index (CSI), precision, recall, and F1 score, defined as in Equations (9)–(14), respectively. Here, true positive (TP) represents accurate predictions of rainfall occurrence, true negative (TN) indicates correct predictions of no rainfall, false positive (FP) represents incorrect predictions of rainfall occurrence, and false negative (FN) indicates incorrect predictions of no rainfall.

(9) $$Accuracy=\frac{TP+TN}{TP+TN+FP+FN}$$

(10) $$FAR=\frac{FP}{TP+FP}$$

(11) $$CSI=\frac{TP}{TP+TN+FP}$$

(12) $$Precision=\frac{TP}{TP+FP}$$

(13) $$Recall=\frac{TP}{TP+FN}$$

(14) $$F1\;score=\frac{2}{\frac{1}{Precision}+\frac{1}{Recall}}$$

In the case of rainfall data, periods without rainfall are typically longer than the period with rainfall. In the study period as well, the no-rainfall period was twice as long as the period in which rainfall occurred. In the case of such imbalanced datasets, the classification performance may appear to be better when predicting no rainfall, as more data are available for the no-rainfall period. If the optimal model is determined based on such a dataset, the detection performance pertaining to rainfall occurrence may deteriorate. Therefore, it is necessary to consider evaluation metrics suitable for imbalanced datasets. Several previous studies have endeavored to tackle the bias problem inherent in imbalanced datasets by utilizing appropriate loss functions for such datasets [40,41,42]. In this study, metrics, such as CSI, which excludes TN when calculating accuracy, and F1 score, which imposes a penalty on no-rain predictions, were selected.

The estimated rainfall intensity was quantitatively evaluated using the root mean squared error (RMSE), *R*^2^, and mean absolute error (MAE), calculated as follows:

(15) $$RMSE=\sqrt{\frac{\sum_{i=1}^{n}{\left(y_{i}-{\hat{y}}_{i}\right)}^{2}}{n}}$$

(16) $$R^{2}=1-\frac{\sum_{i=1}^{n}{\left(y_{i}-{\hat{y}}_{i}\right)}^{2}}{\sum_{i=1}^{n}{\left(y_{i}-\bar{y}\right)}^{2}}$$

(17) $$MAE=\frac{\sum_{i=1}^{n}\left|y_{i}-{\hat{y}}_{i}\right|}{n}$$

where $y_{i}$ is observed rainfall intensity, ${\hat{y}}_{i}$ is estimated rainfall intensity, $\bar{y}$ is average of observed rainfall intensity, and n is the number of samples.

## 3. Results

### 3.1. Feature Value Extraction

**Figure 10:**
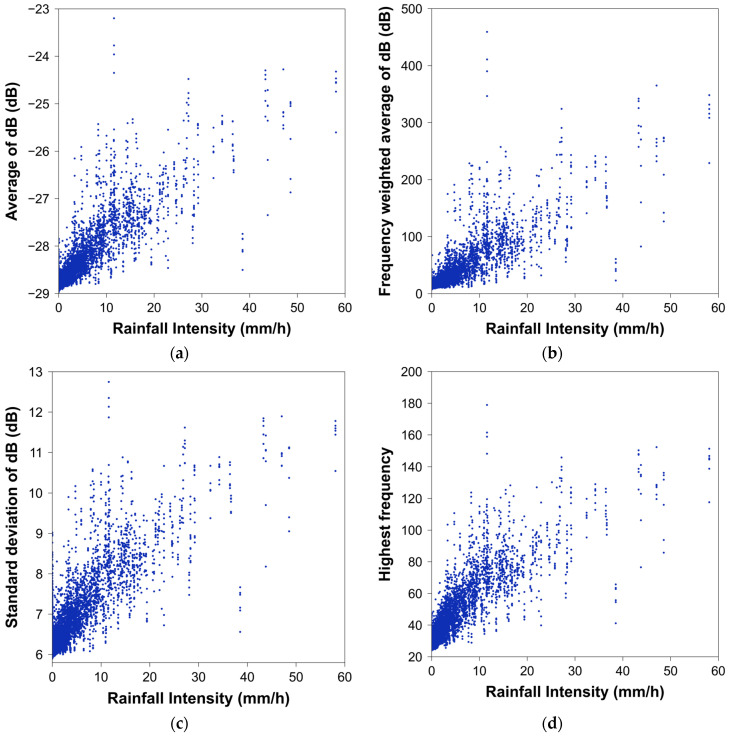
Scatter plot between rainfall intensity and feature values extracted from the 10 s segment acoustic dataset: (**a**) average of dB value; (**b**) frequency-weighted average of dB value; (**c**) standard deviation of dB values; (**d**) highest frequency.

The acoustic data gathered in this study were divided into segments with lengths of 1 s, 10 s, and 1 min. From each segment, the average of dB value, frequency-weighted average of the dB values, standard deviation of the dB values, and highest frequency were extracted. Figure 10 shows each of these feature values extracted from a 10 s long segment.

As mentioned earlier, we found that as the rainfall intensity increases, the frequency distribution of the raindrop sounds extends into the higher-frequency range. Consequently, we observed that the highest frequency increases as the rainfall intensity rises, and both the average and standard deviation of the dB values also increase. This increase is attributed to the emergence of various periodic components with high frequencies in the acoustic data. This pattern is consistent across all four feature values, regardless of the segment duration.

### 3.2. Algorithm Performance for Rainfall Intensity Estimation

The development of the XGBoost-based binary classification model for rainfall detection and the rainfall intensity estimation model was conducted using a workstation equipped with a 12th Gen Intel(R) Core(TM) i5-12400F Processor and 32 GB of RAM. Training the models using only the CPU with 10 s segments required 150 s. The training duration was directly proportional to the number of data points; with the 1 min segments, the training time decreased by approximately six times, whereas with the 1 s segments, the training time increased by approximately ten times.

Using XGBoost, we conducted binary classification to determine the presence or absence of rainfall, employing the four feature values from the frequency domain, as explained in Section 3.2.

**Figure 11:**
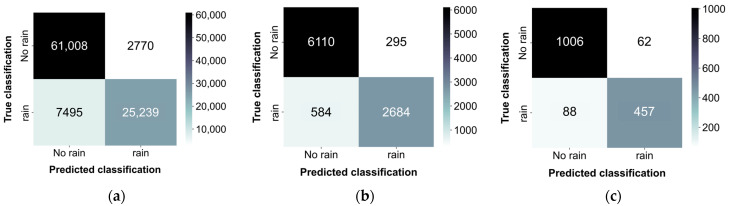
Binary classification results: (**a**) 1 s segments; (**b**) 10 s segments; (**c**) 1 min segments.

Rainfall presence or absence was determined using a threshold of 0.5 mm/h, with observations from the PARSIVEL serving as the basis for this determination. Figure 11 shows the classification results for each segment length as a confusion matrix. Overall, the classification performance was satisfactory. Although certain errors occurred in classifying cases as “rain” when it was not raining, the errors in classifying cases as “no rain” when it was raining were larger. This discrepancy may be attributable to the difficulty in distinguishing low-intensity rainfall from no rainfall. The raindrop sounds of light precipitation may not contain adequately high frequencies to be distinguished from background noise, potentially leading to their misinterpretation as non-rain periods.

Table 3 presents the qualitative evaluation metrics for each segment length. Overall, a similar performance was observed across the segments of three different lengths, with the highest performance achieved with the 10 s segments. But, in terms of recall, higher values were obtained when using the 1 min segments compared to the 10 s segments.

We constructed an XGBoost regression model to quantitatively estimate the rainfall intensity based on the data from the periods identified as having rainfall occurrences from the binary classification results. Table 4 summarizes the final performance metrics for estimating the rainfall intensity. When using 10 s segments, the lowest RMSE of 1.675 mm/h and highest $R^{2}$ value of 0.798 were observed. The lowest MAE of 0.477 mm/h was recorded when using 1 min segments. As mentioned in Section 2.2, it is also crucial to ensure dense time resolution while maintaining a high accuracy in rainfall intensity estimation. Therefore, in this study, the 10 s segments were identified to be the optimal choice, as they provided excellent accuracy along with a finer time resolution.

**Figure 12:**
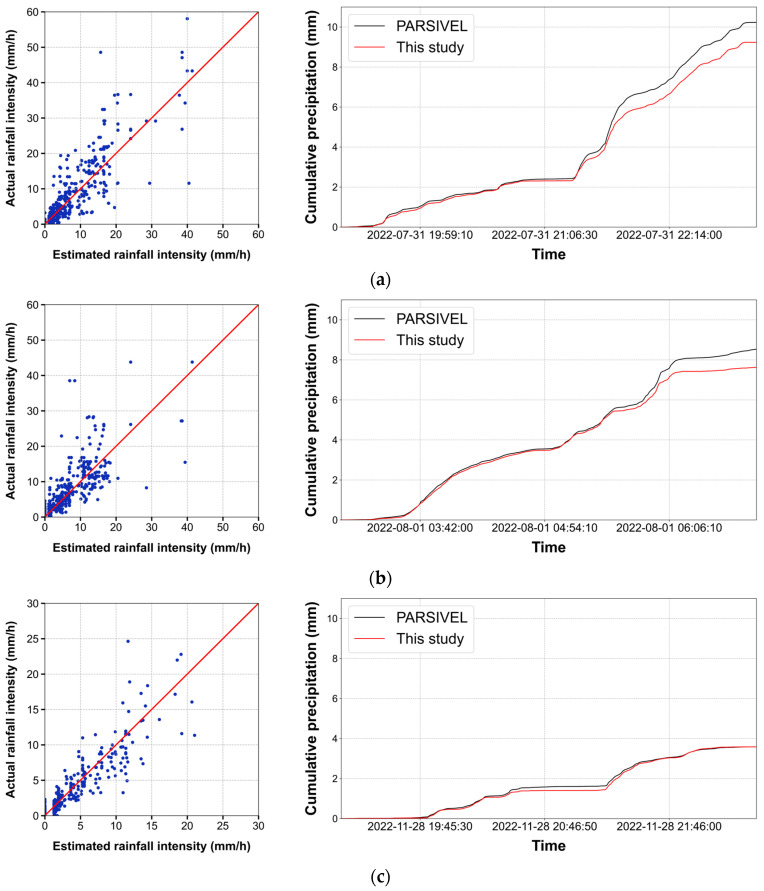
Scatter plot between actual rainfall intensity and estimated rainfall intensity with cumulative precipitation based on estimated rainfall intensity using 10 s segments: (**a**) 31 July 2022, 07:20:30 p.m. to 31 July 2022, 11:03:30 p.m.; (**b**) 1 August 2022, 03:04:00 a.m. to 1 August 2022, 06:48:10 a.m.; (**c**) 28 November 2022, 07:16:00 p.m. to 28 November 2022, 10:27:00 p.m.

Figure 12 shows the rainfall intensity estimated using 10 s segments and cumulative precipitation during three heavy rainfall events with intensities exceeding 15 mm/h during the study period. The values tended to be underestimated, with this underestimation tendency becoming more pronounced with higher rainfall intensities. This observation may be attributable to the imbalance in the training dataset. Notably, in the training dataset with rainfall intensities above 0.5 mm/h, approximately 91.75% of the data points had intensities below 15 mm/h. Consequently, the overall training tendency appeared to be biased toward rainfall intensities below 15 mm/h, as reflected in the underestimation of the higher intensities.

## 4. Discussion

In this study, we aimed to collect consistent rainfall acoustics unaffected by surrounding environmental conditions by placing a condenser microphone inside a waterproof plastic enclosure. This setup was designed to develop a generalized methodology, enabling the application of a rainfall intensity estimation algorithm across regions with varying environmental conditions. To evaluate the generalizability of our proposed methodology, we applied a binary classification model for rainfall detection and a rainfall intensity estimation model, trained on acoustic data collected from the rooftop of Chung-Ang University, to rainfall acoustics acquired under entirely different environmental conditions. As a comparison, another observation site (36°58′57″ N, 127°26′40″ E) located in Jincheon, South Korea, has been considered. It is equipped with ground-based observation instruments such as a two-dimensional video disdrometer, PARSIVEL, and rain gauges. This facility, managed by the Korea Meteorological Administration, serves to compare and verify the performance of operational radars. In contrast to the rooftop of Chung-Ang University, which is situated in an urban area characterized by concrete surrounding environments, the Jincheon site is located in a mountainous region with predominantly vegetative and soil-covered surrounding environments. For the assessment of the generalizability, we installed the acoustic data collection device at the Jincheon site and collected rainfall acoustic data from 05:00 p.m. on 9 August 2023 to 08:00 p.m. on 10 August 2023. Subsequently, we applied the binary classification model for rainfall detection and the rainfall intensity estimation model based on 10 s segments, identified as the most effective in prior analyses, to the rainfall acoustics acquired at the Jincheon site. To evaluate the accuracy of the applied models, we utilized the 1 min interval rainfall intensity data observed by PARSIVEL installed at the Jincheon site.

**Figure 13:**
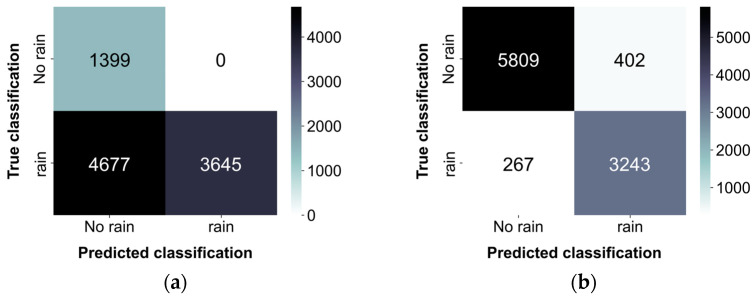
Application results of a binary classification model to detect the presence or absence of rainfall trained on rainfall acoustics from Chung-Ang University to 10 s segment acquisition at Jincheon site: (**a**) rainfall detection threshold established at 0.5 mm/h; (**b**) rainfall detection threshold established at 3 mm/h.

First, we applied the binary classification model for rainfall detection, trained on acoustic data collected at Chung-Ang University, to the rainfall acoustics collected at the Jincheon site to determine the presence of rainfall (Figure 13). Overall, the model trained on rainfall acoustics from Chung-Ang University demonstrated high applicability to the rainfall acoustics collected at the Jincheon site. However, the detection performance significantly degraded for rainfall with an intensity less than 3 mm/h. As shown in Figure 13a, when rainfall events with an intensity of 0.5 mm/h or greater were classified as rainfall occurrences, numerous rainfall events were incorrectly classified as no rainfall. However, as shown in Figure 13b, raising the rainfall occurrence threshold to 3 mm/h resulted in most of the previously misclassified events being correctly classified. The detection performance for rainfall with an intensity of 0.5 mm/h or greater was as follows: accuracy: 0.519, FAR: 0, CSI: 0.438, precision: 1, recall: 0.438, and F1 score: 0.609. For rainfall with an intensity of 3 mm/h or greater, the detection performance improved, with the following values: accuracy: 0.931, FAR: 0.110, CSI: 0.829, precision: 0.890, recall: 0.924, and F1 score: 0.906.

**Figure 14:**
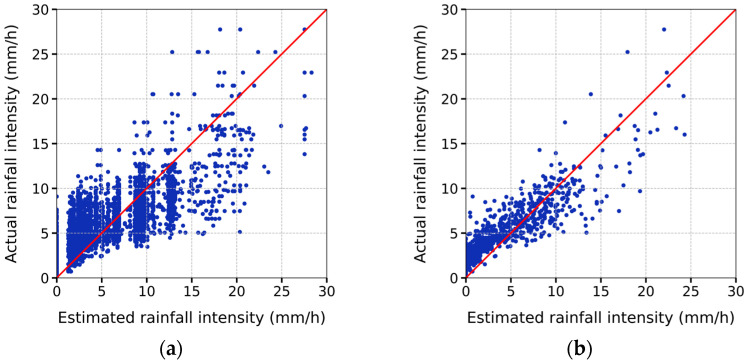
Final rainfall intensity estimation results derived from applying a binary classification model for rainfall detection and a rainfall intensity estimation model, trained on rainfall acoustics from Chung-Ang University, to 10 s segments collected at the Jincheon site: (**a**) comparative result for 10 s interval; (**b**) comparative result for 1 min interval.

The rainfall intensity estimated using the acoustic data collected from the Jincheon site was compared with the rainfall intensity observed by the PARSIVEL installed at the same location (Figure 14). The periods determined to have no rainfall based on the binary classification results were assigned a rainfall intensity of 0 mm/h. The rainfall intensity during the periods determined to have rainfall occurrences was estimated based on the rainfall intensity estimation model trained on rainfall acoustics from Chung-Ang University. Given that the estimated rainfall intensity is predicated on 10 s segments, the resultant rainfall intensity reflects a 10 s observation interval. Conversely, the validation dataset sourced from PARSIVEL entailed data collected at 1 min intervals. Consequently, we adopted the assumption of a sustained, uniform rainfall intensity for each 1 min period corresponding to a single PARSIVEL observation. Accordingly, the PARSIVEL data were converted to 10 s intervals for comparison (Figure 14a). Additionally, the 10 s rainfall intensity estimates, predicated on the 10 s segments, were averaged to 1 min intervals for comparison purposes (Figure 14b). When the rainfall intensity was below 5 mm/h, there was a tendency to underestimate the values. However, based on the overall results, we confirmed the potential for the broad applicability of the proposed algorithm. The accuracy of the 1 min averaged rainfall intensity estimation results yielded an RMSE of 1.851 mm/h, an $R^{2}$ of 0.697, and an MAE of 1.418 mm/h.

Although a slight decline in performance was observed for the weak rainfall intensities, the binary classification and rainfall intensity estimation model, trained on the rainfall acoustics from Chung-Ang University, exhibited considerable applicability to the Jincheon site area. These results underscore the robustness of the methodology employed in this study, as it demonstrated a commendable performance despite the disparate environmental conditions between the training and validation datasets. This highlights notable improvements compared to prior research conducted exclusively within a singular geographic region. Furthermore, the results obtained in this study contrast those of prior studies [18,19,20] aimed at categorizing the rainfall intensity into specific stages based on quantitative assessments. Moreover, notable progress is achieved in the estimation of the rainfall intensity within very short time intervals. However, as our study was conducted on rainfall acoustics for only three short-duration cases and under two environmental conditions, it is necessary to confirm the robustness of our methodology by considering long-term acoustic data collected under more diverse conditions in future research.

However, numerous challenges remain to be addressed to apply the proposed rainfall intensity estimation method to actual rainfall observations. First, the condenser microphone may yield different signal sizes for the same sound depending on their types [43]. The unit of sound size, represented in dB, is a relative measure, defined as the relative ratio of the voltage generated inside the condenser microphone to the reference voltage in response to the sound pressure observed. The size of the reference voltage may vary depending on the type of microphone, and the voltage generated for the same sound pressure may also differ.

Second, this study did not consider the influence of wind, but the differences in rainfall sounds attributable to the effect of wind must be explored. The reduction in rain gauge accuracy due to wind effects is already considered a significant issue. Strong winds can create turbulence around the observational instruments, altering the trajectory of raindrops near the equipment [44]. Similar effects may occur in the acoustic data collection devices utilized in this study. Even raindrops of the same size and falling speed can produce different rainfall sounds depending on the angle of incidence when colliding with the sensor housing and may exhibit different frequency characteristics even at the same rainfall intensity. Therefore, for a more precise estimation of the rainfall intensity, it is essential to mitigate such variability by considering the wind direction and speed in the rainfall environment.

Lastly, addressing privacy concerns is essential. As the proposed method involves acoustic recording technology necessitating the installation of acoustic sensors within public residential areas for effective rainfall observation, privacy issues must be appropriately addressed.

Additionally, to conduct more precise and rapid rainfall observations using the proposed technology, it is essential to establish a wireless transmission environment for the collected data. In the case of acoustic-based rainfall observations, the efficiency can be maximized by deploying observation equipment across extensive areas, given the small data size and cost-effectiveness of the devices. Cloud infrastructure based on wireless data transmission can facilitate verification and calibration through a comparative analysis of observations from adjacent acoustic sensors, enabling the integrated management of data collected from numerous evenly distributed acoustic sensors.

When utilizing acoustic data collection devices, it is also important to consider that temperature can affect the performances of the sensors. During the course of this study, the heat generation of the Raspberry Pi, which constitutes part of the acoustic data collection device, and the consequent performance degradation, was a critical consideration. Moreover, the temperature of the environment surrounding the condenser microphone can alter the characteristics of the collected acoustic signals [45]. Therefore, when estimating the rainfall intensity based on rainfall acoustics, it is necessary to consider temperature as well. It is anticipated that this issue can be addressed in future studies by incorporating a temperature sensor module within the acoustic data collection device.

## 5. Conclusions

This paper proposes a novel approach for rainfall estimation by applying new processing methods based on spectrum analysis and machine learning techniques. An acoustic data collection device was developed by combining a Raspberry Pi with a condenser microphone and installing them within a waterproof housing. This device effectively eliminated background noise, predominantly collecting raindrop sounds. Subsequently, an XGBoost-based binary classification model for rainfall occurrence and rainfall intensity estimation models were developed, using the frequency-domain-derived features from the acoustic signals as input data. Finally, the developed acoustic data collection device and rainfall intensity estimation algorithm based on acoustic data were applied in actual rainfall environments to evaluate their performance. The evaluation was performed in both the monsoon season and non-monsoon season, each involving the collection of rainfall acoustic data. The collected acoustic data were segmented into 1 s, 10 s, and 1 min lengths for comparison, aiming to identify the optimal segment length for rainfall intensity estimation.

The extraction of four feature values from the collected acoustic data was aimed at capturing the increased periodicity in the high-frequency domain with increasing rainfall intensity. The observations indicated that all four feature values increased as the rainfall intensity increased, regardless of the segment length. Overall, the binary classification results for rainfall occurrence were satisfactory. The errors in classifying rainfall as not occurring when it did occur were larger than those in classifying rainfall as occurring when it did not. This discrepancy was attributable to the difficulty in distinguishing the acoustic signals of weak rainfall intensity from those of no-rainfall periods, leading to their misclassification as no rain. The estimated rainfall intensity was similar in trend to the actual values. The best rainfall intensity estimation performance was observed when using 10 s segments. In this case, the binary classification performance was as follows: accuracy: 0.909, FAR: 0.099, CSI: 0.753, precision: 0.901, recall: 0.821, and F1 score: 0.859; and the rainfall intensity estimation metrics were as follows: RMSE: 1.675 mm/h, $R^{2}$: 0.798, and MAE: 0.493 mm/h. The analysis of the cumulative precipitation for three rainfall events with intensities exceeding 15 mm/h indicated the tendency to underestimate the rainfall intensity, which became more pronounced as the rainfall intensity increased. This bias was attributable to the imbalance in the training dataset, where most of the acoustic data corresponded to weak rainfall intensities (below 15 mm/h). Consequently, the training tendency leaned toward these lower-intensity values, resulting in underestimation at higher intensities. Furthermore, during instances of heavy rainfall, thicker water films may develop at the upper section of the acoustic data collection device. The presence of these thicker water films can result in heightened variability in the acoustic signals generated upon raindrop collision with the upper surface of the acoustic data collection device, potentially leading to increased inaccuracies in rainfall intensity estimation. In future studies, it is anticipated that reducing errors attributed to water film formation can be achieved by incorporating a gradient into the upper structure of the acoustic data collection device.

The proposed acoustic-based rainfall estimation method, using small, lightweight, and cost-effective acoustic data collection devices, can generate high-spatiotemporal-resolution rainfall observation data over vast regions. Such data, collected from residential areas, can contribute not only to disaster prevention but also to improving the quality of life through various integrated services. Future research can be aimed at covering larger regions and considering more diverse rainfall events to develop a more generalized and accurate acoustic-based rainfall estimation method.

## Figures and Tables

**Table 1:** Data description.

	Device (Product Name)	Sampling Resolution	Period
Acoustic data	Condenser Microphone (Actto MIC-24)	1/44,100 s	2022.07.31. 03:28 p.m. to 2022.08.01. 01:28 p.m.
			2022.11.28. 04:59 p.m. to 2022.11.29. 11:17 a.m.
Rainfall data	Disdrometer (OTT Parsivel2)	1 min	2022.07.31. 03:28 p.m. to 2022.08.01. 01:28 p.m.
		10 s	2022.11.28. 04:59 p.m. to 2022.11.29. 11:17 a.m.

**Table 2:** Detailed specifications of the PARSIVEL.

Items		Detailed Specifications
Wavelength of optical sensor		780 nm
Measuring area		30 × 180 mm^2^ (54 cm^2^)
Measuring range	Size	0.2~25 mm (32 channel class)
	Fall velocity	0.2~20 m s^–1^ (32 channel class)
Precipitation intensity		0.001~1200 mm h^–1^
Measurement time interval		10 s~60 min
Instrument dimensions (H × W × D)		670 × 600 × 114 mm^3^

**Table 3:** Classification performance for different segment lengths.

Segment Length	Accuracy	FAR	CSI	Precision	Recall	F1 Score
1 s	0.894	0.099	0.711	0.901	0.771	0.831
10 s	0.909	0.099	0.753	0.901	0.821	0.859
1 min	0.907	0.119	0.753	0.881	0.839	0.859

**Table 4:** Rainfall intensity estimation performance according to segment length.

Segment Length	MAE (mm/h)	RMSE (mm/h)	$R^{2}$
1 s	0.554	1.799	0.764
10 s	0.493	1.675	0.798
1 min	0.477	1.705	0.755

## Data Availability

The raw data supporting the conclusions of this article will be made available by the authors on request. The codes used in the study are openly available in GitHub at https://github.com/jinwooklee213/Raindrop_Sound (accessed on 17 June 2024).
